# The hair tales of women of color in Northern Manhattan: a qualitative analysis

**DOI:** 10.3389/frph.2024.1298615

**Published:** 2024-03-15

**Authors:** Chrystelle L. Vilfranc, Lauren C. Houghton, Felice Tsui, Emily Barrett, Adana A. M. Llanos, Kurt Pennell, Desiree A. H. Walker, Micaela Martinez, Beaumont Morton, Peggy Shepard, Mary Beth Terry, Jasmine A. McDonald

**Affiliations:** ^1^ Department of Epidemiology, Mailman School of Public Health, Columbia University Irving Medical Center, New York, NY, United States; ^2^ Columbia University Irving Medical Center, New York, NY, United States; ^3^ Department of Biostatistics and Epidemiology, Rutgers School of Public Health, Environmental and Occupational Health Sciences Institute, Piscataway, NJ, United States; ^4^ School of Engineering, Brown University, Providence, RI, United States; ^5^ Independent Contractor & Patient Advocate, New York, NY, United States; ^6^ We ACT for Environmental Justice, New York, NY, United States; ^7^ Heilbrunn Department of Population and Family Health, Mailman School of Public Health, Columbia University, New York, NY, United States

**Keywords:** endocrine disrupting compounds, hair, hair care products, pregnancy, product use, phthalates, exposure reduction, maternal exposure

## Abstract

**Introduction:**

Exposure to endocrine disrupting chemicals (EDCs), such as phthalates, can negatively impact maternal and child health, contributing to impaired fetal growth, preterm birth, and pregnancy complications, as well as increased downstream risks of cardiometabolic disease and breast cancer. Notably, women of color (WOC) are the largest consumers of personal care products, which are a common source of EDC exposure.

**Methods:**

The Let's Reclaim Our Ancestral Roots (Let's R.O.A.R) Pilot Study developed an educational intervention delivered during pregnancy to promote reduced use of phthalate-containing hair care products (HCPs). This mixed-methods study included: (1) a quantitative analysis and (2) a qualitative analysis of the educational sessions and the semi-structured focus groups to evaluate the factors that influenced the hair care practices and product choices of WOC at various stages of life, including their current pregnancy (hereafter referred to as the hair journey). During the sessions, participants learned about EDCs (with a focus on phthalates), the unequal burden of exposure for WOC, adverse implications of exposure, and exposure reduction strategies. Focus group sessions provided insight into participants' hair journeys from childhood to the current pregnancy and explored factors during their hair product selection process. All sessions were transcribed and imported into NVivo Version 12 for coding and thematic analysis.

**Results:**

A total of 46 individuals were enrolled in the study, and 31 participated in an educational session. This current work synthesizes the qualitative analysis of this study. We identified two important life stages (before and after gaining agency over hair care practices and product choices) and three dominant themes related to HCP use: (1) products that impacted the hair journey, which involved all mentions of hair products, (2) factors that influenced the hair journey, which included individuals or entities that shaped participants' hair experiences, and (3) the relationship between hair and sense of self, where sense of self was defined as the alignment of one's inner and outer beauty.

**Conclusion:**

The themes intersected and impacted the participants' hair journey. Cultural integration was a sub-theme that overlapped within the dominant themes and participants discussed the effect of traditions on their hair experiences.

## 1 Introduction

Endocrine disrupting chemicals (EDCs) are a group of compounds that mimic and disrupt different pathways in the endocrine system (1). EDC exposure *in utero* is associated with an increased risk of pre-term birth, obesity, and neurodevelopmental disorders for the fetus and an increased risk of chronic diseases and breast cancer for the mother (2, 3). Fetal EDC exposure has been more specifically linked to: cognitive deficits (4), obesogenic fetal programming (5), and negative impacts on testis development and future male fertility (6). Therefore, EDC exposure during pregnancy is a critical period during which EDC exposure will impact both fetus and mother. For many, personal care product use is a source of EDC exposure, as individuals often begin use of personal care products early in life and exposure often continues daily over the life course (7). Among the common EDCs found in personal care products, phthalates are often added to hair care products (HCPs) for a variety of enhancing purposes, including providing scent to products (8, 9). Phthalates are anti-androgenic (10, 11) and have been shown to interfere with hormone regulation as they can interact with both estrogen- and androgenic-related biological pathways (3, 8, 9). Exposure during critical windows of susceptibility for breast cancer includes windows of life where the breast tissue undergoes rapid changes (prenatal, pubertal, pregnancy, lactating and menopause transition). Exposures during the pregnancy window also have intergenerational effects on mother and fetus (2–6); therefore, interventions designed for reducing exposure during this time are potentially impactful.

EDC exposure during pregnancy may be particularly harmful for WOC due to their increased exposure to EDC-containing products, as compared to their counterparts (12–14). Phthalate metabolites have been found to be significantly higher among women of color (WOC) (3). This finding is unsurprising as WOC, especially Black women, are among the most frequent consumers of personal care products including HCPs which is in part due to varying hair textures among WOC (15, 16). In pregnant women, greater personal care product use is associated with higher concentrations of urinary phthalate metabolites and urinary metabolite concentrations vary by the type of personal care product (17–21). While there are studies analyzing EDC exposure concentrations in pregnant women, as well as studies that have analyzed their attitudes and intention to modify their use of personal care products during their pregnancy (22), very few studies focus on pregnant WOC.

Environmental exposure interventions have been established as an important method to promote better health outcomes, yet few studies consider HCP use and behaviors over the life course. These interventions promote environmental health literacy where environmental exposure literacy is combined with health literacy to inspire individuals to make informed decisions, take steps to reduce health risks, and work to protect the environment (23). Studies have highlighted and connected the issues of environmental justice and beauty product chemical exposures among WOC (24, 25); therefore, focusing on WOC, especially pregnant WOC, may have an impact on both mother and child, impacting multiple generations. Consequently, we designed an educational intervention study to reduce phthalate exposures during the pregnancy/postpartum window and increase environmental health literacy, with the long-term goal to improve fetal and maternal health.

The conceptualization and study design of the Let's Reclaim Our Ancestral Roots (Let's R.O.A.R) pilot study were realized through trusted partnerships with community leaders which included WE ACT for Environmental Justice and a breast cancer advocate and champion. WE ACT originated as a Harlem-based organization in 1988, created to tackle local environmental racism, but has grown to tackle environmental justice on a national level. The breast cancer advocate (DAHW), with leadership roles within organizations like the Young Survival Coalition which addresses the unique needs of young adults affected by breast cancer, served as the study's community scientist. The overall objective of the Let's R.O.A.R pilot study was to understand and determine if an educational intervention during pregnancy could decrease the use of phthalate-containing HCPs among pregnant WOC. We performed a quantitative analysis for this pilot study, which will be published separately. However, we were equally interested in performing a qualitative assessment to understand the impact this intervention would have on the behavior of our participants. Therefore, we explored the hair journey of WOC. We defined the hair journey as the relationship WOC have had with their hair starting with long lasting and impactful memories, their hair care journey over time, and overall sentiments regarding their hair. Within this journey we defined agency as the moment that WOC gain freedom or control of the decisions regarding their hair. Here, we report the qualitative phase of the study and participants' insights into their hair journey, including hair product use and perceptions regarding EDCs.

## 2 Materials and methods

### 2.1 Participants

We recruited pregnant WOC in the Northern Manhattan region in 2021 who were on average 31.37 ± 3.30 weeks pregnant. We consented a total of 46 study participants, 4 were recruited virtually through Instagram and 44 from an Obstetrics & Gynecology (OB/GYN) clinic. Our research staff contacted individuals who expressed interest in the study to assess their eligibility. Participants were eligible for the study if they: (1) were at least 18 years of age, (2) residents of Northern Manhattan or other New York City boroughs, (3) self-identified as a WOC, (4) were in their second or early third trimester of their pregnancy, and (5) had a smartphone and/or computer for zoom sessions. Forty-six individuals were eligible, consented, and enrolled in the study. Our study population was multicultural and racially and ethnically diverse. Fifty-two percent of women were born outside the United States (U.S.) and 78% identified as Hispanic, Latina, or of Spanish origin. Among those of Hispanic ethnicity (*n* = 36), 36% identified as Hispanic Black, 39% as Hispanic other, 8% as Hispanic White, 9% self-reported as additional race origins, and 8% refused to report on race. Ten participants identified as Black and not of Hispanic, Latina, or of Spanish origin (*n* = 10). Of the 46 individuals, 31 participated in an English or Spanish educational intervention discussing the adverse implications of using phthalate-containing HCPs, as well as a focus group session.

### 2.2 Educational intervention sessions and focus groups

Our team facilitated ten 1-hour educational intervention sessions that were delivered via Zoom and attendance varied between 1 and 5 participants. The first part of the educational session included introductions followed by a member of our team prompting the attendees to share a brief background of their HCP use. We wanted to learn what participants could recall about their earliest experiences with their hair, including when and what products were being used in their hair during the period before they were able to make decisions regarding their hair journey. We asked the participants the following questions: (1) How far along are you in your pregnancy? (2) What is the earliest age you remember having someone putting product in your hair? (3) At what age did you take agency over your hair care routine? Then participants tuned into the educational video and PowerPoint. Following these presentations, participants participated in a brief question and answer segment and debriefing from the project coordinators.

Our study was modeled after a few key theoretical frameworks. Finn and O'Fallon's Environmental Health Literacy framework proposes that providing knowledge or information to individuals about their environmental exposures will empower them and inspire them to make more health-conscious decisions with regards to how they interact with the environment and the potential exposures (23). We also incorporated Marshall Ganz's Public Narrative: Self, Us, Now framework which suggests grounding the audience to be sure they are engaged, using methods such as storytelling, listening, and reflecting (26). The study sessions were designed to provide knowledge and engage the participants to have awareness and inspire action. Additional frameworks and concepts included discrete decision-making and consumer theory (27, 28) to understand their journey with their hair and what motivates their behaviors and product choices.

We conducted three 1-hour focus group sessions which were designed to gather feedback on the behavioral intervention, to discover an individual's hair journey from childhood to pregnancy, and to probe the major factors that have an effect on their current product selection process. Initially, the focus group sessions were delivered as stand-alone sessions no more than eight days after the educational intervention portion. However, given our observations that the participants were challenged by attending two separate sessions, the semi-structured focus group guide was incorporated into the end of the educational intervention sessions for seven of the ten total sessions. Given that all focus groups followed educational sessions, we do not expect the timing of the focus group to have influenced participants' responses. The questions posed included: (1) How has your self-care routine changed from before pregnancy to now? (2) What factors do you consider when selecting hair care products? (3) How does your hair influence how you perceive yourself?

All participants were compensated for meeting the various milestones throughout the study, including for attending the educational intervention session.

### 2.3 Data processing and management

We transcribed all English (*n* = 7) and Spanish (*n* = 3) educational intervention sessions and three stand-alone focus group sessions. A native Spanish speaker reviewed the Spanish session transcripts and translations for accuracy and to ensure cultural nuances were addressed and reflected appropriately. We uploaded all transcripts into NVivo Version 12 to manage and analyze the data through thematic analysis and coding.

### 2.4 Qualitative data analysis

We used thematic coding to analyze the transcriptions (29). The researchers (CLV, LCH, and JAM) compiled a list of common elements identified during the review of five of the ten educational session transcripts to create an initial codebook of parent codes (the overarching and most representative elements), child codes (corresponding themes that stemmed from a parent code), definitions of each code, and examples of text for each code. To ensure the clarity of the origin of each code, codes that were developed based on the responses from questions the research team posed in the educational and focus group segments regarding hair journey, products, and sense of self were labeled as the “Deductive” codes. Codes that emerged while reviewing the transcripts were labeled as “Inductive” codes. Three of the ten educational sessions were accompanied by separate focus group sessions until the research team decided to incorporate the focus group segment within the remaining seven educational sessions. Independently, CLV coded the stand-alone focus group transcripts (*n* = 3) and the remaining educational transcripts (*n* = 5). CLV identified additional parent and child codes that arose inductively and modified the codebook accordingly, which was discussed with LCH and JAM before being incorporated into the codebook. We used five of the ten sessions to create the initial codebook and assess saturation. Saturation was reached after coding nine of the transcripts. The final codebook contained 9 parent codes (over-arching themes) and 33 child codes (each of which corresponded to a parent code).

To identify the potential themes, one researcher (CLV) manually performed concept mapping to demonstrate the relationships between the parent codes based on the coding results. Additionally, we used the data analysis tools available through NVivo, including the hierarchical clustering analysis to repeat and validate the relationships produced manually. Together, these analyses produced graphical representations of the most coded parent codes and mapped their relationships with one another. Based on the clustering of the parent codes, the potential dominant themes were identified and discussed among the team (CLV, LCH, and JAM).

## 3 Results

### 3.1 Women of color and their hair journey throughout the life course

We characterized hair care practices over the life course to frame our analysis, including: (1) the phase *before* gaining agency to make choices with regards to hair care and styling practices, (2) the phase *after* gaining agency, and (3) the *current* phase during their pregnancy. Our study revealed that the WOC who were participants in our study had very keen memories of their earliest encounters with hair products (i.e., before agency) and they were able to describe a range of factors that they take into consideration when choosing hair care products now (i.e., after agency).

**Figure 1:**
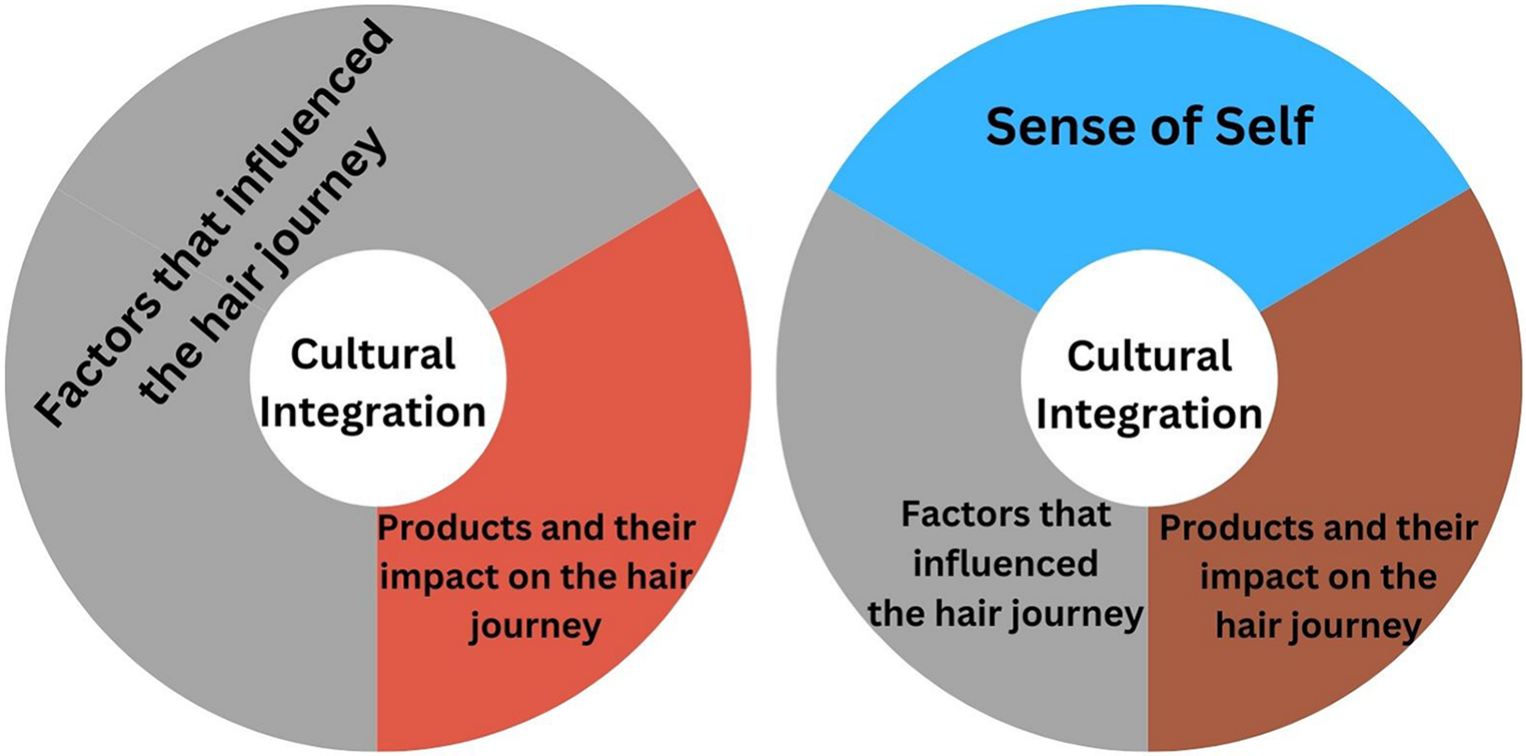
Identifying the dominant themes. Three dominant themes that impacted the hair journey of our participants were identified as: products and their impact on the hair journey, factors that influenced the hair journey, and the relationship between hair and sense of self. Cultural integration was an overlapping subtheme for all dominant themes.

There were three dominant themes, which collectively impacted the participants' hair journey, before and after gaining agency: (1) products that impacted the hair journey, (2) factors that influenced the hair journey, and (3) the relationship between hair and sense of self (Figure 1). Cultural integration emerged as a sub-theme that impacted the three dominant themes.

### 3.2 Before agency

#### 3.2.1 Products and their impact on the hair journey

Before agency, participants did not necessarily make choices or provide input on what HCPs might be used in their hair. The purchasing of HCPs was often the responsibility of caregivers and/or guardians. Many participants recalled specific products used in their hair before they had agency. Participants were able to think back to specific ages hair product use began, product types, and even sensory-related memories such as the smell of specific products that were being used in their hair. A few participants could not recall a time in their life when products were *not* used in their hair. One participant, U.S.-born 33-year-old, stated:

*“I have been using hair products since I was probably just out of infancy, as long as my hair was long enough, there was product in it.”*

Cultural integration was a prominent sub-theme that could not be ignored, as it impacted the participants' hair journeys before agency. Living outside of their family's country of origin, many participants' hair journey relied on culturally traditional practices. There were numerous examples of how culture determined the types of products used in their hair before they gained agency. One said:

*“I'm actually Indian. So, we are really big on like coconut oil and this other oil that's called Amla oil and that's supposed to be really good for our hair… That was put on my hair when I was young. So, that's something that our parents all gave us when we were young, but it's like completely natural.”*

Participants recalled the importance of homemade products for hair care during early periods of their life. Homemade hair masks were especially popular among the women from non-U.S. backgrounds, including culinary ingredients, such as avocado, mayo, and butter. A U.S.-born Dominican 22-year-old recalled her mother's homemade hair masks:

*“My mom, she is Dominican, so like I grew up like her, always like doing like avocado hair mask and stuff like that. Like, she made her own stuff when she felt like my hair was dry.”*

Participants also recalled their caregivers struggling to style and maintain the participant's hair. During the period before agency, their caregivers often considered the participant's hair texture or hair type as a factor for choosing the products they would use in their hair. Participants were aware of the differences in their hair texture in comparison to their caregivers or other individuals in their household. One participant stressed that her mother's hair was different from her hair because her mother was white. She recalled her mother considering their difference in hair textures when purchasing products:

*“I never told her what to get me, but we have like completely opposite [or] different hair like her hair is thin and straight and then my hair is really curly and thick. So, I think she would get stuff specifically for me, because it was completely different.”*

Another multiracial participant recalled her Mediterranean father unintentionally highlighting her hair because of his frequent application of baby oil to her hair.

*“I was raised in a mixed household, and he was a single dad at the moment, and he didn't know what to do with my curly hair… he would soak it in baby oil, and it probably got highlights because it was being fried.”*

#### 3.2.2 Factors that influenced the hair journey

Before agency, caregivers influenced HCP use and practices. Many women reflected on the lack of agency they had when they were younger regarding their hair and that the decisions being made concerning their hair were the responsibility of their primary caregivers, which were predominantly mothers. One recalled:

*“I remember my mom putting in products on my hair since I was a little baby…I remember being little, like two, three, and going to like Easter Sunday and like her putting Lottabody in my hair.”*

Most of our participants alluded to their caregivers' decision-making being impacted by external factors ranging from ethnic culture, salon culture, and/or special events. For example, Dominican participants recalled their mothers or other family members as salon owners and/or frequenters of salons. For many Dominican participants, Dominican salons appeared to be integral to the Dominican hair experience.

*“With like my family that my aunts or my mother, you know, since I am Dominican, having a hair salon is like, you know, there is always a family member with one. So, my aunt had a hair salon… So, I never did my own hair. They had someone usually like wash my hair and do the rollers.”*

With close and trusted family members as salon owners, many participants were brought into salon culture early in life. Moreover, caregivers trusted the salon professionals with the participants' hair care and maintenance. Beauticians at the salon were permitted to make reversible and irreversible decisions from the use of temporary hair styling products to the use of permanent hair relaxers. One participant remembered:

*“When I was about 10 someone [at] the salon actually relaxed my hair because she said that my hair was too thick, and she couldn't handle it and I was 10. So, I am not going to argue with an adult. And my grandma was there. She said okay. So, I was relaxing my hair for about five years just because that is how it started. Like I wasn't really, I didn't really have a choice.”*

It was evident that most participants did not have agency over the decisions being made to their hair; therefore, their caregivers primarily regulated their hair journey early in life.

### 3.3 After agency

Study participants recalled the moments they gained agency over their hair care from purchasing their hair product, to deciding on a new style, or coloring their hair for the first time. For most participants, they gained agency during their pre-teen or early teenage years. During this period after agency, the themes of products that impacted the hair journey, factors that influenced the hair journey, and cultural integration remained key, but the participant's relationship between hair and sense of self became an equally important theme (Figure 1).

#### 3.3.1 Products and their impact on the hair journey

After agency, participants chose what products to use but trusted various sources to guide their product choices. A few of our participants admitted that while they had agency and were now the decision-makers regarding their hair care practices, they did not experience having to do their own hair until the COVID-19 pandemic, as they often frequented salons which were temporarily shut during this time. A 22-year-old participant stated that:

*“I used to go to the salon a lot. So, I don't think that is me taking care of my hair. I started going to the salon, like on my own when I was like 14, 15. And then recently, like during quarantine, I started taking like real good care of my like natural hair, like on my own, like picking my own products and stuff. So, I want to say when I was 20, I took agency over my hair completely.”*

Participants who were born outside of the U.S., or whose guardians were born outside of the U.S., mentioned being sent products from their countries of origin, from shea butter to certain shampoos. This was especially common among Dominican and African participants, establishing an intersection between product choice and one's culture. Even after agency, we found that for a few of our non-U.S. born participants, culture shaped their choices regarding their hair, especially when it came to product choices. It was evident that these participants held onto effective and trustworthy practices of their cultures and/or countries of origin, including integrating products from “back home” into their haircare regimen. A 33-year-old Liberian participant reported using “donut grease” or Shea Butter on her children's hair which she gets from family members when they return from traveling “back home.” A 30-year-old Dominican participant said:

*“I remember when I used to live in Dominican Republic…what we usually do there [is] like natural products, like made of carrots, avocado and things like that. And at the moment those are the type of products that I try to use. My mother sends them to me from Dominican Republic.”*

#### 3.3.2 Factors that influenced the hair journey

Family members and salon professionals remained key influencers for many of our participants after gaining agency. For some of our participants, the opinions of family members remained valuable. Many women continued in salon culture, entrusting their haircare to professional stylists and medical providers. During one focus group session, when asked if most of the products used in their hair currently or in the past were first introduced at a salon, the participants replied in the affirmative. In fact, purchasing products recommended by professionals was not unusual for some of our participants, especially those who described different hair-related conditions. One of our Dominican participants, 29 years old, mentioned:

*“But if a dermatologist would tell me, no matter the price, this is going to work for your hair loss or this is going to work for the type of hair, I will get it. I will get it.”*

After gaining agency, influencers also included friends and social media. Product advertisement in the past largely consisted of radio and television commercials, which likely impressed the caregivers of our participants during the before agency stage. Today however, social media has become one of the primary and most powerful sources for product advertisement. One participant mentioned:

*“Now with social media, it plays a big role in our daily lives. So, it's like, you can see somebody that has natural curly hair or kinky hair, so okay, maybe I should give it a try. I'm a perfect example. I saw Tracee Ellis Ross's and I was like, I might as well go to Ulta to check up on it.”*

One of our African participants reported struggling with terrible dandruff and recalled purchasing a line of products she heard about from an African social media influencer who also struggled with dandruff. The participant reached out to the influencer:

*“She used to have dandruff a lot. And this product was very helpful, so that's how I contacted her. And then she gave me the lady's number and then I talked to the lady. So, that's how I went along, paid for it, 80 dollars for the whole set.”*

A few women reported learning about potential risks of long-term exposure to personal care products with harmful chemicals through media platforms. A 31-year-old Dominican participant recalled her daughter watching a Tik-Tok video about harmful chemical exposures in hair products. It inspired her and her daughter to investigate a mobile phone application that provided information on chemical exposures in hair products. She recalled:

*“I was noticing lots of hair when she would take a shower and wash her hair and it would be hair everywhere or she would be clogging our pipes all the time. So, then I started looking into it and I noticed that the product that we were using was really high in like dirty chemicals. Like it was on the Think Dirty app, it was coming out red.”*

#### 3.3.3 The relationship between hair and sense of self

**Figure 2:**
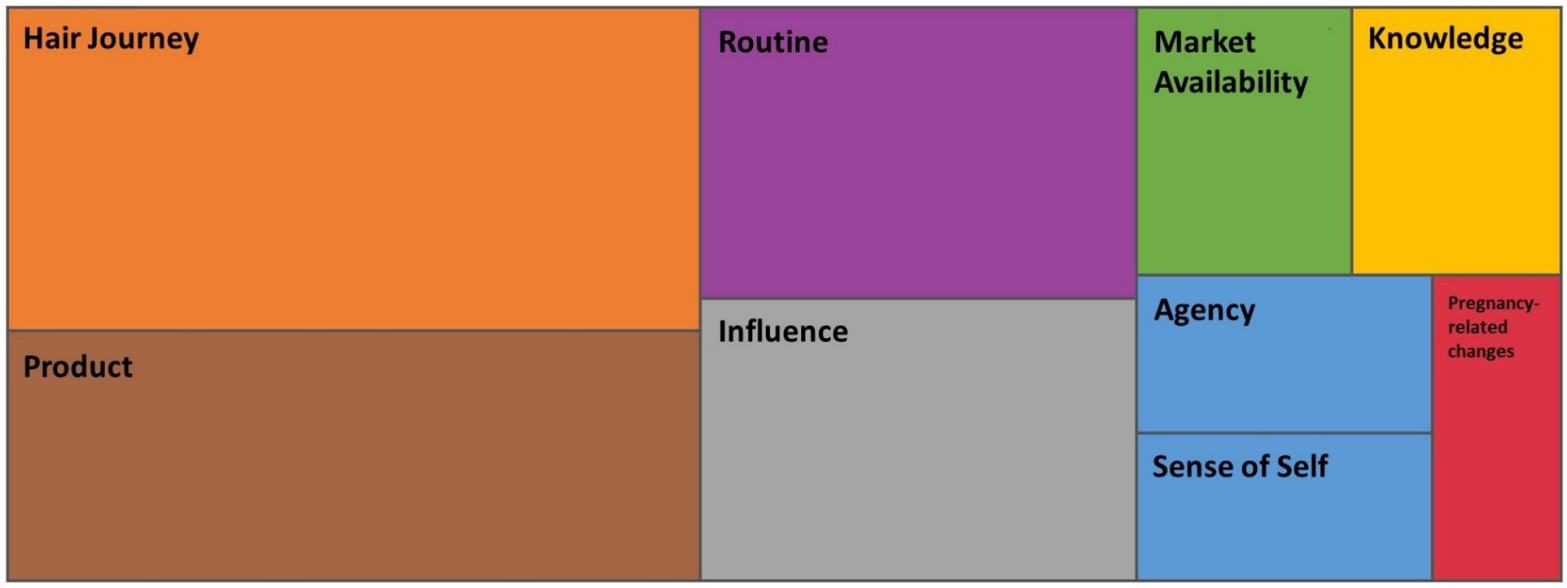
NVivo hierarchy tree clustering. Hierarchy tree map chart developed using NVivo coding software. Each box, represents a parent code, and the size of the box, represents the most frequently coded parent codes. The positions of the parent codes relative to the surrounding parent codes demonstrate potential relationships with the surrounding codes.

Establishing the relationship between hair and sense of self appears to begin during the period after gaining agency (Figure 2). Once the participants had agency over their hair journey, we noticed discussions of being able to better align their inner and outer beauty. The participants began to make clear connections between how they felt about their hair (their outer beauty) and how they felt about themselves (inner beauty and self-esteem) with regards to when they were responsible for choices regarding their hair. Participants expressed that doing their hair has always helped brighten their moods, cheered them up, or made them feel beautiful. These discussions were not observed during discussions regarding the “before agency” period. It was evident that the relationship to their hair and their sense of self began forming at the time they began to have agency and remained important throughout their life.

Familial influences stretched beyond choosing products that impacted the hair journey, to also influencing participants' relationship between hair and sense of self, and how they felt their inner beauty and outer beauty aligned. Despite their control over their hair journey, participants remarked that they had family members who would criticize how they styled their hair, or when they made major changes to their hair. One 33-year-old U.S.-born Dominican participant stated that:

*“I've learned how to manipulate my curls so that you can still wear it natural, and it still looks like presentable…you only feel pretty, attractive or good when your hair was pretty straight and that took a toll on me… I learned how to diffuse properly and how to properly do it so that I can feel confident in my own curls my own way… But now I'm grateful that I took some time to learn and try, because it can be scary, especially I'm Dominican as well and it can be scary going out into our world where everyone sees the girl with [an] image of perfection. Even family members, they'll be like, oh, do your hair, you look like a bruja, that's like a witch or whatever it is.”*

Social pressures, including beauty standards, were identified as additionally having an effect on our participants after they had agency over their hair choices. A 33-year-old U.S.-born Hispanic participant stated:

*“I should be able to feel good in my natural state, you know, and I shouldn't have to keep transforming myself just to fit in. So, it was a hard realization, but I had to break away from that because I know that I can't be chained to the beauty industry and I can't be a chain to the salon, you know, just to feel good about myself.”*

There was also a clear connection between cultural integration and the relationship of one's hair journey and their sense of self that was present after gaining agency. A participant of Mexican and Indigenous descent shared:

*“Yeah, really, I feel ugly like it's a matter how short I've cut it and I just wanted powerful, feminine and I don't know how to I guess express or let them know. So I prefer having my long hair, straight long black and I come from an indigenous background and we don't normally cut our hair…I learned more about my father's [Indigenous] side … there's a whole community out there who find strength in their hair… and it made me feel like, I don't know, welcomed, belonging. So, I've read more and got into more of spirituality of the idea of hair and strength and what it meant to the indigenous community and that's what I hope to pass down to my children so they can understand how hair is and what it means.”*

#### 3.3.4 Pregnancy

Participants did not view pregnancy as a distinct window with regards to hair care practices. Most of our participants did not acknowledge pregnancy as an important period until they were prompted during the focus group portions of the study. Only then did these participants describe changes they had made to their hair care and styling decisions during their pregnancy.

Many participants noted that while pregnancy may have caused changes to their hair, comfortability, and mood, their routines changed more due to the COVID-19 pandemic than pregnancy. Yet, a considerable number of our participants reported prioritizing care of their hair in some form, especially in attempts to empower their sense of self. One participant mentioned:

*“If I'm feeling off and I do my hair, that will change my entire mood in a matter of a couple of hours. The health of my hair, prior to pregnancy, throughout pregnancy, it's extremely important. For me, to be able to do my hair and feel beautiful that's everything. That's [the] number one thing.”*

A few women reported learning about potential risks of long-term exposure to personal care products with harmful chemicals through media platforms prior to attending our educational intervention. However, during the end-of-session participant feedback, many participants reported gaining knowledge about the potential health risks from personal care product exposures. Many were especially appreciative of gaining this knowledge during their pregnancy, as they reported considering the new information with regards to choosing personal care products for their babies. The empowerment these participants expressed gaining with the provided knowledge was a major goal of this pilot study.

## 4 Discussion

Pregnancy is a window of susceptibility for women's health and lowering chemical burden could have positive impacts on the health outcomes of both mother and baby. Through focus groups and educational sessions with WOC we explored whether pregnancy would be a key phase that contributed to participants' hair journey, especially regarding their hair product choices. We spoke to WOC during their pregnancies, and few mentioned making major changes to their hair routine during this time. Many gained agency over their hair practices much earlier in their lives. Thus, there is a need to identify the most appropriate timing to effect change while considering the intersection between biological windows of susceptibility and the most culturally salient windows.

The Let's R.O.A.R pilot study followed Finn and O'Fallon's health literacy framework and Marshall Ganz's Public Narrative: Self, Us, Now framework (23, 26). The health literacy framework objectives promote the acquisition, comprehension, application, evaluation, and use of knowledge in terms of environmental health literacy to improve health outcomes for individuals and their communities (23). The public narrative framework engages the audience by incorporating methods such as storytelling, listening and reflecting (26). Let's R.O.A.R aimed to understand the awareness and perceptions of HCP use on psychosocial adjustment, health, and risk behaviors of pregnant WOC, and normative beliefs of hair and their identity. Therefore, we provided an educational intervention and obtained urinary data, but it was also equally important to allow WOC psychosocial space for conversations regarding their hair experiences during such a pivotal point of their lives, like their pregnancy.

The French PREVED Intervention Study educated pregnant women on methods for identifying and choosing alternatives to food pollutants, environmental pollutants, and personal care products (30). The group sought to measure and compare urinary metabolite concentrations among the participants who received the intervention, as well as quantify various psychosocial dimensions (i.e., gauging their self-esteem, risk perception, and their expectations of a healthy baby). Like the Let's R.O.A.R study, the PREVED study focused on evaluating psychosocial changes of pregnant participants, including self-esteem, risk perception, and the level of concern for EDC exposures. However, the PREVED study was not racially diverse and may not capture the psychosocial dimensions experienced by WOC living within the American standard of beauty. While there are other racially diverse environmental exposure intervention study models for pregnant women, they do not include psychosocial or qualitative assessments (31, 32). The Let's R.O.A.R study aimed to promote social and cultural awareness of EDCs and reduction of EDC exposure during critical windows of susceptibility. The Let's R.O.A.R pilot study aims to synergize the importance of a quantifiable intervention with psychosocial dimensions that offer an open space for shared stories and bonding with the intention of empowering pregnant WOC movement towards action.

The relationship between hair and sense of self arose as a particularly important theme during the period after participants gained agency over their hair journey. The transitionary period for when participants recalled first gaining agency remained a vivid memory where participants came into their agency for various reasons. A few mentioned that it became more convenient for them to start doing their own hair as their caretaker(s) was no longer available. Other participants revealed that their transition into agency over their hair journey occurred in pre-adolescent or early adolescent ages. Given puberty through adolescence is a crucial window of susceptibility to environmental exposures, learning of EDC exposures and health prior to gaining agency could be effective in changing behavior as evidenced in the *HERMOSA* intervention study. In the *HERMOSA* study, measurement of urinary metabolite exposures in young Latina girls pre- and post-intervention demonstrated that EDC exposure was reduced upon education and being provided alternative, cleaner personal care products (33). A caveat to this period is that full agency may not be achievable presenting challenges in the routine implementation of healthier options.

Corroborating others (34, 35), we also observed that a sense of self was connected to women's hair care styling practices as it was where inner beauty matched outer beauty. Many of our participants reported considering hair products based on function and ability to manipulate their hair, i.e., defining curls. The fixation of HCPs on hair manipulation can likely be traced back to the fixation of society on labeling hair as “good hair” (straighter/longer/finer) vs. “bad hair” (tightly coiled/kinky/coarse), which has had negative psychosocial consequences on WOC, especially Black women (24, 36, 37). The marketing of products to promote beauty and certain societal beauty standards, rather than bodily health, may be a structural barrier to having the ability to make the healthier choice, further contributing to environmental beauty injustice. Ideas of beauty and structural barriers should be considered when designing interventions; otherwise, education is not enough if it makes unrealistic expectations of individuals when they are facing upstream forces. Moreover, ideas of beauty and structural barriers must also be considered in context to the target population.

Participants also expressed narratives regarding family as a major influential factor in the hair journey. Prior to gaining agency, most participants admitted to not having much of a say in their hair journey, such as the products being used, and decisions being made. Participants fully trusted and/or depended on the authoritative individuals in their life, which often included family. After gaining agency, some of our participants described family influences continuing to impact their hair journey, whether it was internalizing comments their family would make regarding their hair, referrals from family members to use certain products, or salon referrals. Our findings further validate prior studies that demonstrate the existence of a strong relationship between familial and caregiver influence and a Black woman's hair journey (38, 39).

Our participants expressed narratives that were unique to intersectional identities such as the multiracial narrative. For some of our participants, their multiracial and/or multiethnic identities, were an important aspect of their hair stories. One poignant story was told by a participant of Mexican and Indigenous heritage who described her childhood surrounded by her Mexican family members. She often felt pressured to style and cut her hair similarly to that of those around her. However, as she got older and learned more about her Indigenous roots and Indigenous hair practices, she became inspired to retain her length and felt a sense of strength as she continued to grow her hair. Studies discuss the shift in multiracial individuals' identities, including their physical appearances over time (40); this participant's story and that of other participants of multiple races and/or ethnicities further illustrates what Pauker, Lukate and Foster referred to as the malleability of the multiracial identity and how the intersectional identities of those of multiple races and/or ethnicities are experienced within one's self and outward (34, 40). In our study, this is where we saw strong connections between cultural integration, sense of self, and the overall hair journey.

The cultural integration subtheme was especially prominent amongst our Dominican participants with respect to salon culture. The Dominican beauty salon industry in New York City is well documented and our Washington Heights/Inwood area is known to have a Dominican salon on nearly every block, practically serving as a “cultural staple” for Dominican neighborhoods in New York City (41). Candalerio argues that the beauty shop helps shape the connection between culture and identity. We found this to be true, as our participants discussed their first Dominican salon visit as a rite of passage. Some even recalled the age they were first able to go to the salon without their guardian. The salon was where they first experienced the transformation of their hair to their culture's more acceptable standards of beauty. For one of our participants, the salon was where she experienced her first negative feeling towards her hair as the stylists gave her a hair relaxer to make her hair more manageable. Many Black and mixed-race women grapple with their sense of self and their relationship to their hair and beauty. This is due to historical and present-day societal pressures, where lighter skin and straighter hair has been established as close to whiteness, thus closer to beauty, femininity, and acceptability (34). In 2018, Mitchell and colleagues' multiracial and multiethnic qualitative analysis found that most individuals judge their “typicality” (how similar they perceive themselves to be to their ethnic-racial group) and their “atypicality” (differences compared to their ethnic-racial group) based on a number of factors including hair, skin color, and facial features (42). Mitchell specifically suggested that their Latinx participants judged themselves as atypical amongst other Latinx individuals due to their appearance, which was usually a result of adopted stereotypes such as being Latinx with darker skin and “black hair” (42).

Our findings reflect a specific place and time. Participant demographics represent the demographics of Northern Manhattan and the surrounding boroughs, with 78% of Hispanic, Latina, or Spanish descent, of which 44% of our participants identified as Dominican. We conducted this study during the COVID-19 pandemic, which forced us to move to a virtual engagement. While the virtual setting may have limited meaningful engagement with some participants, it also made the study more accessible for some participants, particularly those who were in the final trimester of their pregnancy and reported being uncomfortable or feeling sick. Future studies could purposefully sample across various age groups, abilities, gender identities, and pregnancy trimesters to delve into more specific questions pertinent to subgroups.

Our qualitative study impressed credibility as investigator triangulation was used during the coding process by involving several researchers as research team members. These individuals were involved in addressing the organizational aspects of the study and the process of data analysis. While data was coded by one researcher, the initial working codebook and the themes were analyzed and established by three different researchers. Analysis also included the generation of a manual concept map followed by a comparison with the NVivo cluster analysis. When the interpretations of the researchers differed, the discrepancies were thoroughly discussed until the most suitable interpretation and best representation of the meaning of the data was established.

Our study is not without limitations. With the racial and ethnic makeup of our participants, it is important to note the origin, race and ethnicities of the individuals who collected and analyzed the data during this study. It is important to note that all three of the researchers who led the data analysis (CLV, LCH and JAM) are not Hispanic migrants or of Hispanic origin, unlike the majority of our participants. However, the researchers would identify themselves as culturally aware and experienced with working with communities of Hispanic origin due to their engagement with these communities through their research work, community outreach, and their community partnerships. The educational intervention and focus group facilitators were women of color, two African American women and one native Spanish-speaking Hispanic woman. One of the facilitators is a cis-gender Southern Black woman with 4C textured natural hair (JAM). Another facilitator is a Puerto-Rican native Spanish speaker with wavy hair, mostly worn straightened (AR). A third facilitator is an African American woman and cancer survivor whom, after losing her hair during her cancer treatment, continues to-date to shear her hair, donning a bald head (DAHW). Thus, while blind spots exist, hair commonalities and differences between the participants and facilitators may have encouraged respondents to either, be more open or be more reserved, in sharing their hair journey.

**Figure 3:**
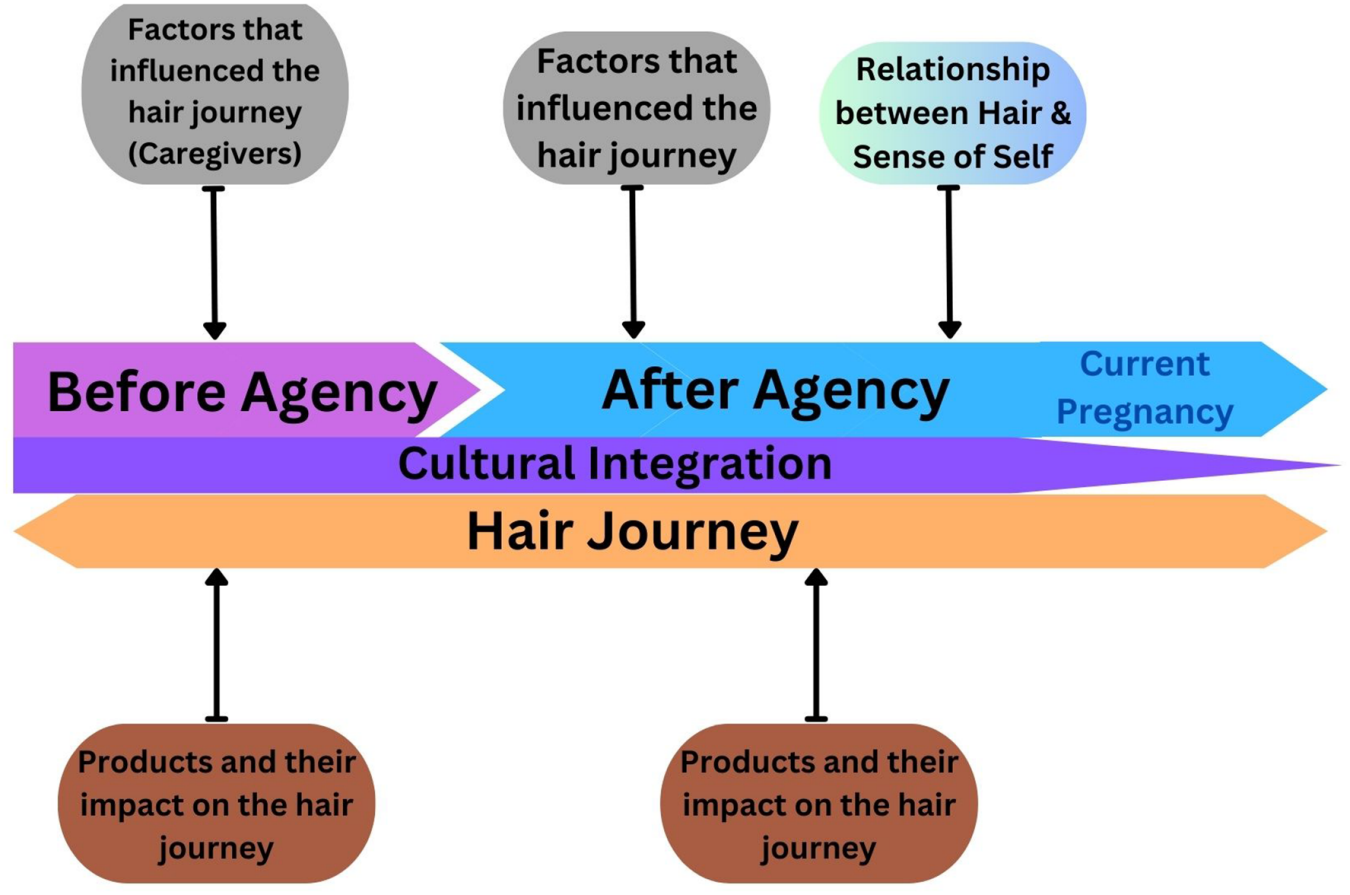
Illustrative study schematic. There were two major stages in the hair journey: “before agency” (pink) and “after agency” (blue), with the current pregnancy of the participants falling under “after agency.” Products and their impact on the hair journey (brown) and factors that influenced the hair journey (grey) were major factors during both stages of the hair journey; however, “sense of self” began during the period “after agency.” Cultural integration (purple) remained an overlapping subtheme throughout the entire hair journey (orange double arrow).

We found that products that impacted the hair journey, factors that influenced the hair journey, and the relationship between hair and sense of self were important contributing factors to the hair journeys of the WOC in our study. These themes were interconnected in that the factors that influenced the hair journey often informed decisions and/or inspired what products participants purchased or how participants styled their hair (Figure 3). These factors had lasting impacts on participants' sense of self and/or their relationship to their hair. This coincided with previously published studies where participants reported receiving negative remarks about their hair or wearing their hair in its natural state from their maternal figures and/or other female family members (43, 44). Cultural integration was evident with regards to the products purchased and/or used, and how participants styled their hair to better connect with their heritage and strengthen their sense of self. These factors were crucial in the telling of hair stories and experiences throughout these WOCs' lives. Through speaking with WOC during pregnancy, it was apparent that there is an opportunity and need to include qualitative psychosocial dimensions to inform culturally salient and inclusive interventional studies. However, our study also provided further insight that qualitative psychosocial dimensions can further our understanding on the commonalities and the unique factors that influence oneness with inner and outer beauty for WOC living in a society where mainstream beauty standards are not inclusive.

## Data Availability

The raw data supporting the conclusions of this article will be made available by the authors after all publications using the raw data or publications being supported by the raw data have been published, without undue reservation.
